# Unilateral Eales’ disease a case report

**DOI:** 10.22336/rjo.2017.27

**Published:** 2017

**Authors:** Andreea Nicolcescu, Carmen Mocanu, Loredana Dinu, Andrei Olaru, Mara Ionete, Dima Alin Stefanescu

**Affiliations:** * Department of Ophthalmology, Clinical Emergency Hospital, Craiova, Romania; ** Department of Ophthalmology, University of Medicine and Pharmacy of Craiova, Craiova, Romania

**Keywords:** Eales, recurrent floaters, focal laser

## Abstract

**Introduction.** Eales disease is an idiopathic peripheral vascular occlusive disease characterized by inflammation, ischemia, and retinal neovascularization and is hallmarked by recurrent vitreous hemorrhages and vision loss.

**Case report.** We present a case of a 48-year-old female with recurrent floaters and decreased vision in her right eye. The onset of symptoms was in 2007 when a diagnose of retinal vasculitis was made. She had no accompanying systemic signs and symptoms and no history of ocular trauma or previous tuberculosis infection. The eye condition was managed only with intermittent focal laser treatment, because the general treatment with steroids was not efficient and poorly tolerated. After the laser treatment, the visual acuity completely recovered and there was no recurrence of vitreous hemorrhage.

The case particularity was the unilaterality after 9 years from the onset.

## Introduction

Eales disease is an idiopathic peripheral retinal vasculopathy that affects the peripheral retinal vasculature, leading to retinal non-perfusion or ischemia, retinal neovascularization and vitreous hemorrhage. There may be perivasculitis, phlebitis, dilated aneurismal changes shunt vessels and even macular edema [**1**]. 

## Case report

We present the case of a 48-year-old myopic female with recurrent floaters, and decreased vision in her right eye.

From the medical history, we found that the onset of the symptoms was in 2007, when a diagnose of retinal vasculitis was made. She had no accompanying systemic sign and symptoms and no history of ocular trauma or previous tuberculosis infection. The eye condition was managed only with intermittent focal laser treatment because the general treatment with steroids was not efficient and poorly tolerated.

The symptoms reoccurred in November 2015 when she was admitted in the Eye Clinic with vitreous hemorrhage in her right eye. 

At presentation, her best corrected visual acuity was 0,5 for the right eye and 0,9 for the left eye, with a spherical myopic correction (-3,75). By the applanation of tonometry, the intraocular pressure was 15 mmHg for both eyes. On the external examination and slit-lamp examination, the findings of the anterior pole were within normal limits. The fundus of each eye was examined after a pharmaceutical mydriasis with 1% tropicamide and 10% phenylephrine hydrochloride ophthalmic solution.

Fundus examination of the left eye was normal (**Fig. 2**).

**Fig. 1:**
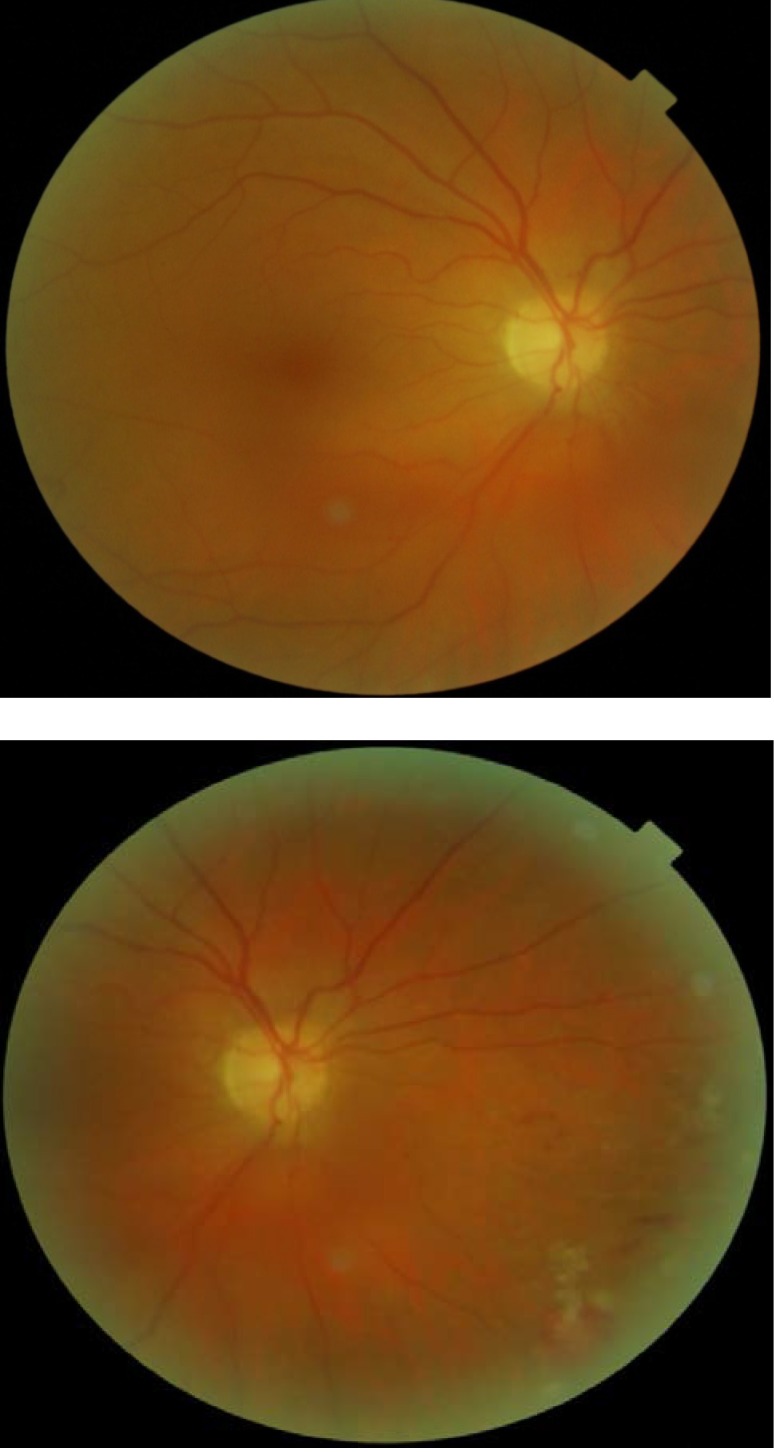
Right eye: Vitreous hemorrhage and the retina – blurry vision

**Fig. 2:**
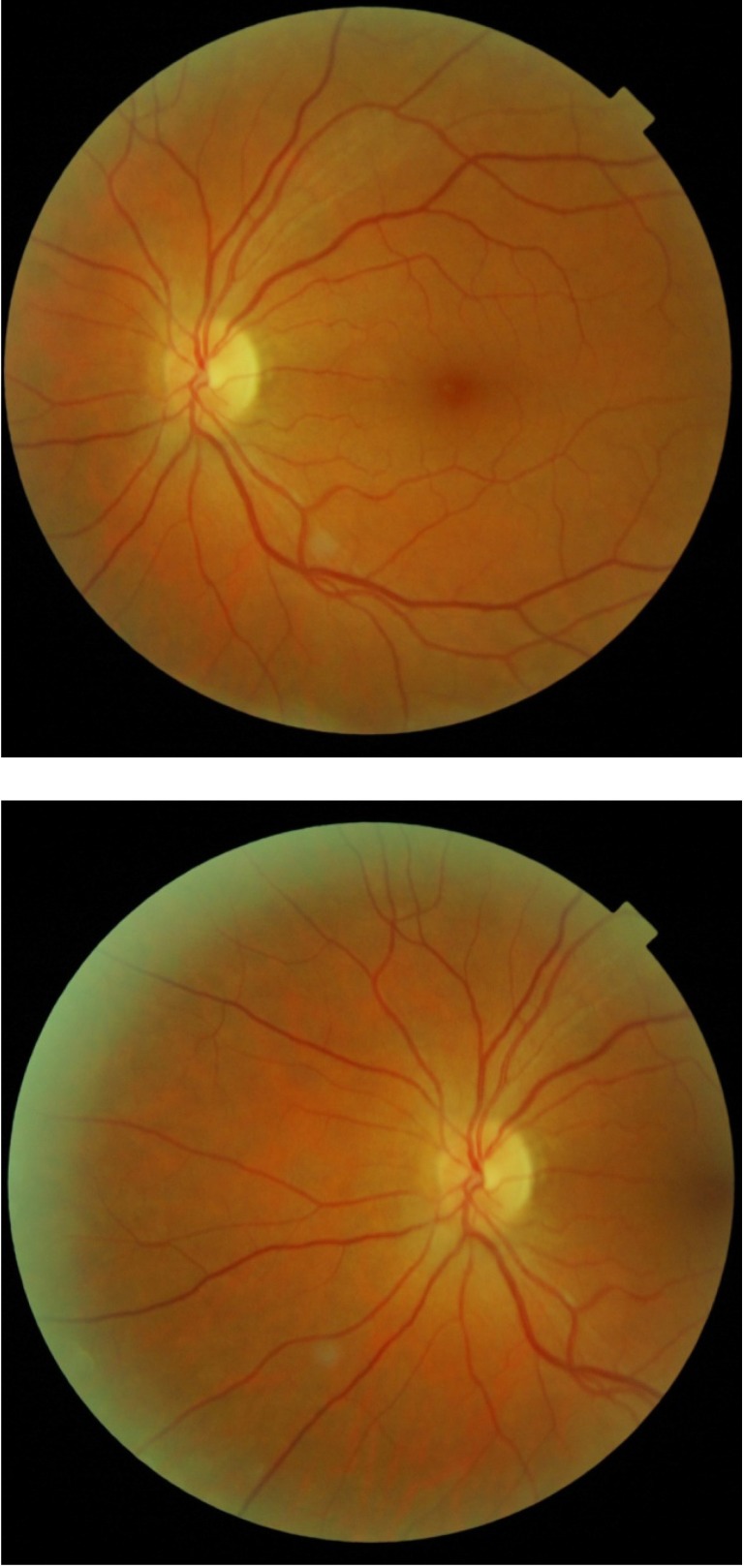
Fundus examination of left eye is normal

**Fig. 3:**
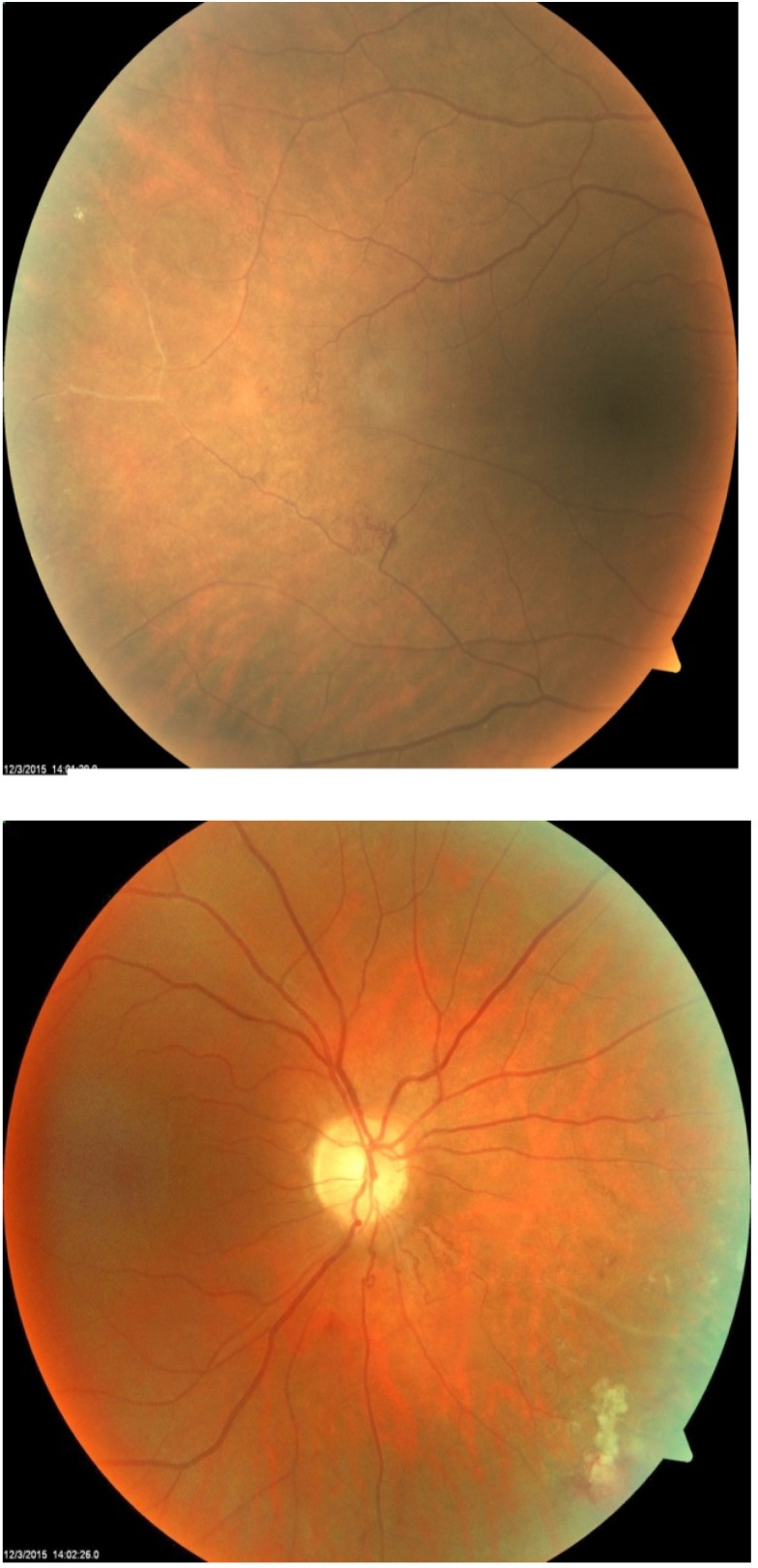
Right eye after the vitreous hemorrhage is gone: NVE, fibrovascular proliferation, peripheral non-perfusion, collaterals, vascular sheathing, peripheral BRVO

After the vitreous hemorrhage was gone, at the fundus examination of the right eye we noticed: retinal neovascularization, fibrovascular proliferation, peripheral non-perfusion, collaterals, vascular sheathing, and peripheral BRVO (**Fig. 3**).

**Fig. 4:**
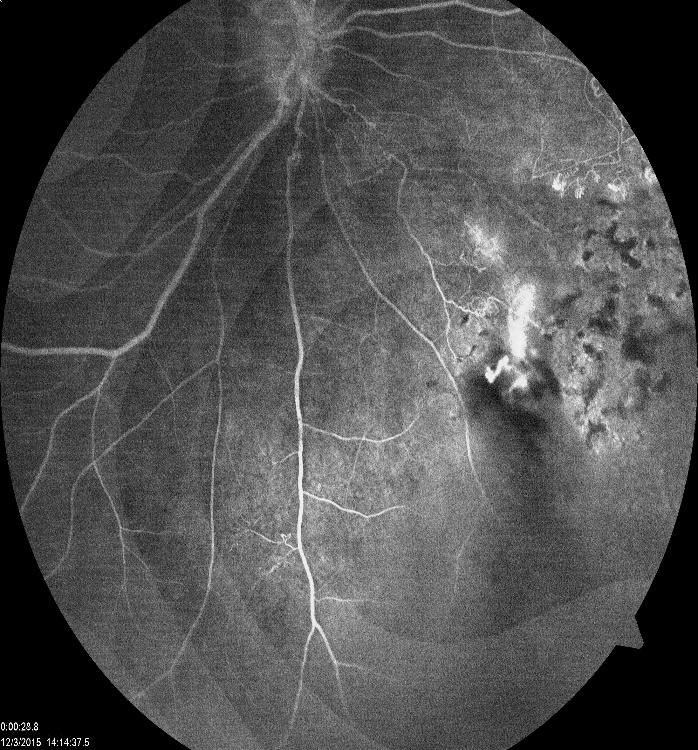
FA on the right eye: peripheral non-perfusion; retinal neovascularization with leakage

**Fig. 5:**
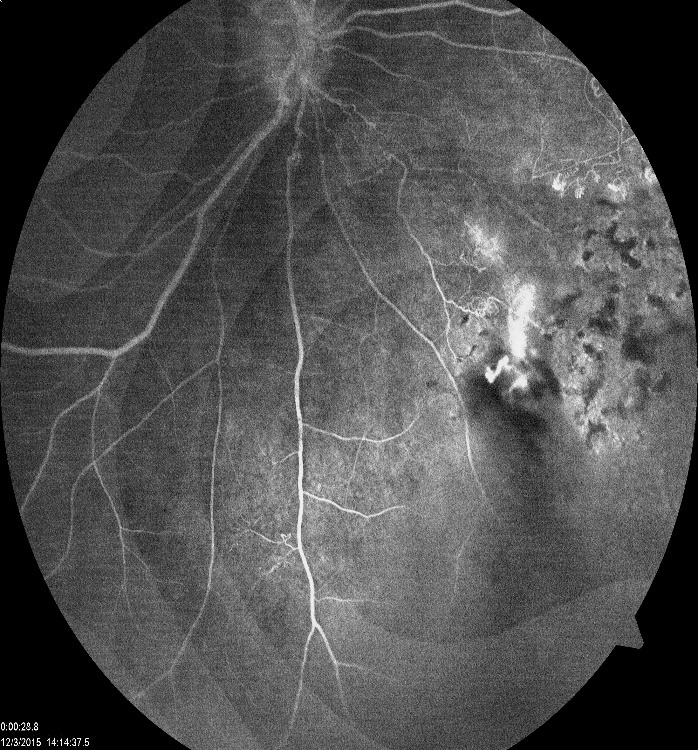
FA on the left eye is normal

Fluorescein angiography revealed peripheral non-perfusion and retinal neovascularization with leakage in the right eye (**Fig. 4**) and was normal for the left eye (**Fig. 5**).

Other etiology was excluded, because of the possible causes of this condition. Hematologic diseases were ruled out as possible causes of the condition. Normal chest X-ray and negative tuberculin PPD test ruled out the tuberculosis infection. Systemic lupus erythematosus was ruled out (normal antinuclear antibody, rheumatoid factor or C-reactive protein, negative C3 C4).

The raised erythrocyte sedimentation rates (28/ 46 mm) and elevated cholesterol (331 mg/ dl) were not specific enough for a particular explanatory condition either.

Based on the medical history, the fundus aspect and paraclinical investigation, and the diagnosis of Eales disease was established in the right eye.

Focal laser treatment of the areas of new vessels was performed in the right eye, FA- guided. The evolution was favorable after the laser treatment, the new vessels regressed, the visual acuity completely recovered and there was no recurrence of vitreous hemorrhage (**Fig. 5**).

**Fig. 6:**
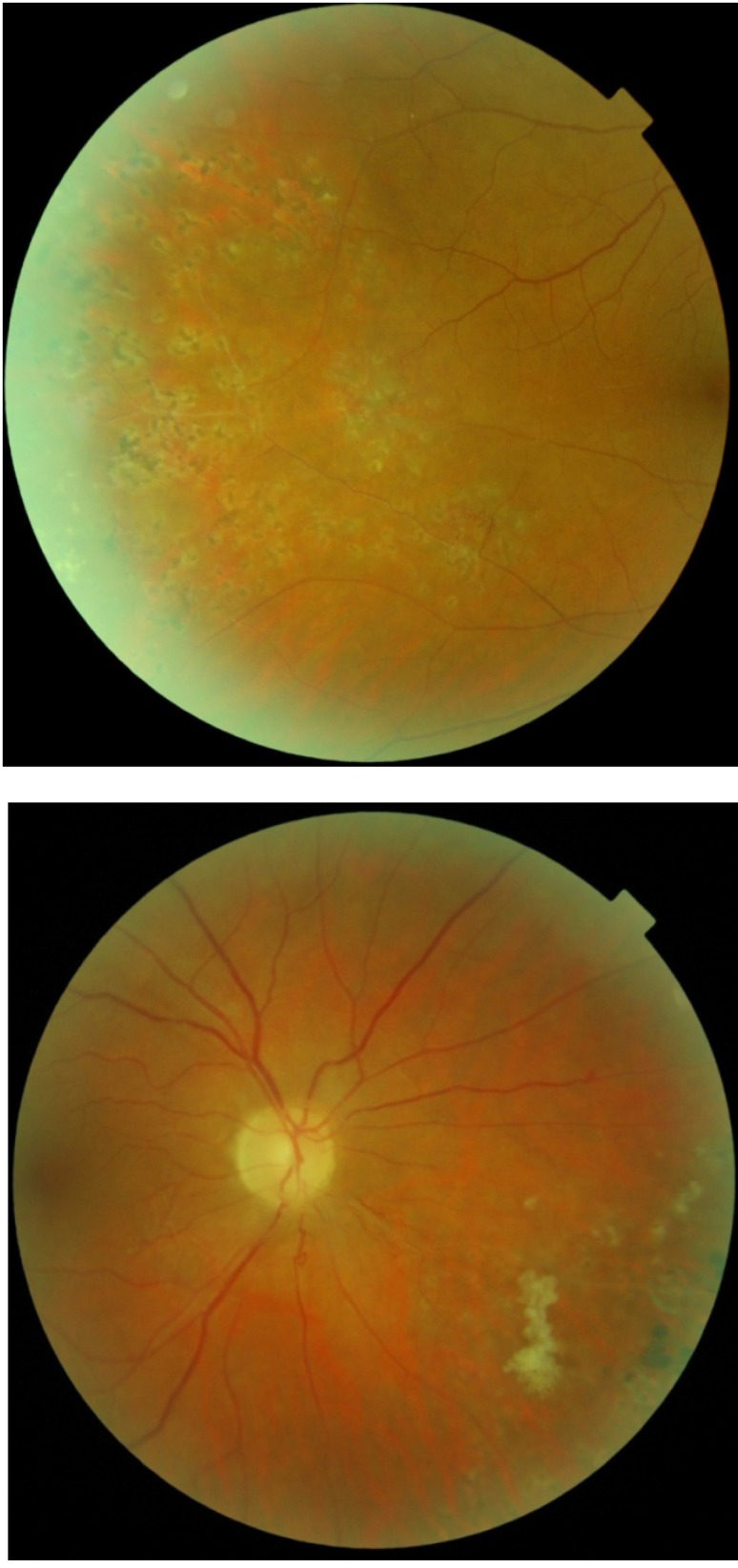
Right eye: aspect after laser treatment

The patient had a follow up period; at every 3 months, the reoccurrence of new vessels in the right eye and eventually the involvement of the left eye being monitored.

## Discussions

Eales Diseases have the name of Henry Eales, who, in 1880, described five young men with recurring vitreous and retinal hemorrhages associated with constipation and epistaxis [**4**].

The etiology is unknown, no definite cause has been found for Eales disease, being considered idiopathic. It tends to occur in males of Asian origin in their second decade of life (20-40 years old) [**3**]. Moreover, it has been related to tuberculosis exposure and hypersensitivity to tuberculoprotein and tubercle bacilli have been identified in pathology specimens [**6**]. Other immune-mediated mechanisms, such as predominant T-cell involvement, have also been demonstrated through experimental studies [**5**]. Associations have also been made with retinal S-antigen and HLA B5, DR1, DR4, and patients with autoimmune disease. Support has been found for oxidative stress, as increased accumulation of lipid peroxide, increased carboxy methyl lysine, decreased glutathione, and decreased vitamins C and E. Similarly, increased vascular endothelial growth factor (VEGF) results have been found in retinal neovascularization [**2**]. 

Peripheral retinal vessels first became inflammated, sheathed, and then occluded. The loss of perfusion leads to retinal neovascularization that causes recurrent vitreous hemorrhages. It is initially asymptomatic, but then progresses through general stages of the disease [**2**].

Early stage, inflammatory: various stages of periphlebitis may be found, venous dilatation and perivascular exudates can be found as well [**2**].

Middle stage, ischemic: characterized by capillary ischemia, demarcation between perfused and non-perfused zones is marked by veno-venous shunts, venous beadings, and microaneurysms [**2**].

Late stage, proliferative: neovascularization occurs at the junction between perfused and non-perfused retina that leads to recurrent vitreous hemorrhages with or without retinal detachment [**2**].

Eales disease tends to occur peripherally, but macular edema and disc neovascularization may also occur. Patients may present with decreased vision, photopsia and floaters unilaterally or bilaterally [**2**].

Fluorescein angiography shows leakage of the sheathed vessels, retinal vascular tortuosity and telangiectasias, shunt vessels, venous stasis, capillary non-perfusion, retinal neovascularization [**2**].

Eales disease is a clinical diagnosis of exclusion, chest X-ray; the Montoux tuberculin shin test might be helpful (because the Mycobacterium tuberculosis DNA has been found in the vitreous of patients with Eales disease). Hemoglobin electrophoresis can assess the sickle cell retinopathy. VDRL is useful for syphilis and anti-nuclear antibody (ANA); it can also indicate systemic lupus erythematosus. Serum angiotensin converting enzyme and a chest X-ray can point to sarcoidosis [**2**].

In the early or inflammatory stage, oral prednisone (1-1,5 mg/ kg tapered over 6-8 weeks) is recommended in patients with involvement of two quadrants, with maintenance dose (15-20 mg per day for up to 2 months) if needed [**2**].

However, in patients with macular edema or involvement of three quadrants, intravitreal triamcinolone acetonide has shown improvement in visual acuity [**7**].

Non-steroidal immunosuppressive agents are reserved for those in whom steroids are ineffective or contraindicated [**2**]. Intravitreal anti-VEGF therapy may be successful with vascular nonperfused and retinal neovascularization; however, its effects may cause vitreoretinal contraction [**8**].

In patients with exposure to tuberculosis, anti tubercular therapy can be administered for 9 months, but it is reserved for patients with massive infiltration, nodule formation, and venous obliteration [**2**].

For patients with a later stage disease retinal neovascularization, laser scatter photocoagulation (peripheral pan-retinal photocoagulation) in the areas of retinal non-perfusion induces regression of neovascularization. When optic disc neovascularization is present, panretinal photocoagulation laser is recommended. Pars plana vitrectomy is necessary for non-resolving vitreous hemorrhage and/ or retinal detachment (whether tractional, rhegmatogenous, or combined). Endolaser treatment may be applied at the time of surgery. Vitrectomy for non-resolving vitreous hemorrhage should be performed no later than 6 months following the onset of hemorrhage [**2**].

Eales disease is a diagnosis of exclusion. In the absence of other systemic conditions, peripheral retinal inflammation and recurrent vitreous hemorrhages are important defining features.

Laboratory tests are not sensitive or specific for Eales disease but may be helpful in detecting systemic causes of retinal vasculitis or peripheral retinal non-perfusion.

With the proper treatment, the prognosis of Eales disease is good. The major cause of visual loss is recurrent vitreous hemorrhages. Complications of neovascularization, such as retinal detachment and neovascular glaucoma, may contribute to significant vision loss, however, blindness is rare, and vision less than 20/ 200 occurs in less than 1% of the patients [**2**].

## Case particularity

Eales disease is considered a bilateral disease in up to 90% of the patients, although symptoms often present unilaterally [**2**]. The particularity of this case is the unilaterality after 9 years from the onset.
